# Asymptomatic breast implant rupture detected in pregnancy: a case report and a management pathway

**DOI:** 10.1093/jscr/rjag247

**Published:** 2026-04-14

**Authors:** Nghĩa M Nguyễn, Trinh T Võ, Sơn B Trần, Hiền M Nguyễn, Hiển T Phạm

**Affiliations:** Department of Plastic and Reconstructive Surgery, Hanoi Medical University, No. 1 Ton That Tung Street, Kim Lien Ward, Hanoi 10000, Vietnam; Department of Plastic Reconstructive and Aesthetic Surgery, Hoe Nhai General Hospital, Branch 2, No. 34, Alley 53, Tan Ap Street, Hong Ha Ward, Hanoi 10000, Vietnam; Department of Plastic and Reconstructive Surgery, Hanoi Medical University, No. 1 Ton That Tung Street, Kim Lien Ward, Hanoi 10000, Vietnam; Department of Plastic Reconstructive and Aesthetic Surgery, Hoe Nhai General Hospital, Branch 2, No. 34, Alley 53, Tan Ap Street, Hong Ha Ward, Hanoi 10000, Vietnam; Department of Plastic Reconstructive and Aesthetic Surgery, Hoe Nhai General Hospital, Branch 2, No. 34, Alley 53, Tan Ap Street, Hong Ha Ward, Hanoi 10000, Vietnam; Department of Plastic Reconstructive and Aesthetic Surgery, Hoe Nhai General Hospital, Branch 2, No. 34, Alley 53, Tan Ap Street, Hong Ha Ward, Hanoi 10000, Vietnam; Department of Plastic Reconstructive and Aesthetic Surgery, Hoe Nhai General Hospital, Branch 2, No. 34, Alley 53, Tan Ap Street, Hong Ha Ward, Hanoi 10000, Vietnam

**Keywords:** breast implant, rupture, pregnancy, MRI, pathway management

## Abstract

Breast implant rupture during pregnancy is rarely reported, and physiologic breast changes may obscure clinical detection. Management should prioritize maternal–fetal safety with rupture-related concerns. We report a gravida 3 woman with a 7-year-old textured silicone implant placed subpectorally in whom left intracapsular rupture was incidentally suspected during a breast ultrasound performed alongside routine antenatal assessment at 32 + 5 weeks’ gestation. She remained asymptomatic, with no palpable abnormalities, inflammatory changes, or systemic signs. After multidisciplinary counseling, she was managed expectantly with scheduled clinical reviews and defined return precautions. Magnetic resonance imaging (MRI) was deferred during pregnancy because results were unlikely to change antepartum management. Postpartum MRI confirmed left implant rupture. Elective surgery and implant exchange seven months postpartum was uncomplicated. This case supports ultrasound-led, safety-netted observation until postpartum, and reinforces routine surveillance and preconception implant checks for women planning pregnancy.

## Introduction

Breast augmentation with implants is among the most commonly performed aesthetic operations worldwide. In the 2024 ISAPS Global Survey, an estimated 1 658 615 breast augmentations were performed globally; in Vietnam, 16 920 procedures were reported (8.4% of all cosmetic surgical procedures), placing it among the country’s most frequently performed aesthetic operations. As implant-based breast augmentation becomes increasingly widespread, clinicians across specialties—including obstetrics—will inevitably encounter implant-related concerns during pregnancy.

Implant rupture is an important late complication of silicone breast augmentation. Long-term Kaplan–Meier-based data report a 10-year rupture rate of ~13.0% per patient and 7.7% per implant [1]. Many ruptures are clinically silent or present with nonspecific changes in contour or firmness [2, 3], making diagnosis based on physical examination alone unreliable and often necessitating imaging confirmation.

Pregnancy adds complexity in both assessment and decision-making. Physiologic breast changes may obscure subtle contour changes [4, 5], while concerns about fetal safety and breastfeeding can increase anxiety and perceived urgency for interventions. However, pregnancy-specific management guidance for suspected implant rupture in pregnancy is limited, and practical guidance describing how to balance reassurance, surveillance, and escalation is inconsistently presented. We therefore report a case of an asymptomatic pregnant patient with incidentally suspected intracapsular rupture and outline a practical pathway with predefined return precautions that enables safe deferral to postpartum while minimizing unnecessary imaging, surgery, and psychological distress. This also has broader relevance for counseling and planning in women with breast implants prior to pregnancy.

## Case report

A 27-year-old gravida 3 woman with a singleton pregnancy was referred at 32 + 5 weeks’ gestation a patient-requested breast ultrasound during routine antenatal care incidentally suggested left implant rupture. She was asymptomatic and denied breast pain, swelling, rapid breast enlargement, nipple discharge, a palpable mass, or systemic symptoms. She had undergone bilateral cosmetic breast augmentation in 2018 via an inframammary incision with round textured silicone implants placed subpectorally (reported 378 cc; Allergan). She reported no postoperative complications and denied breast trauma or manipulation.

**Figure 1:**
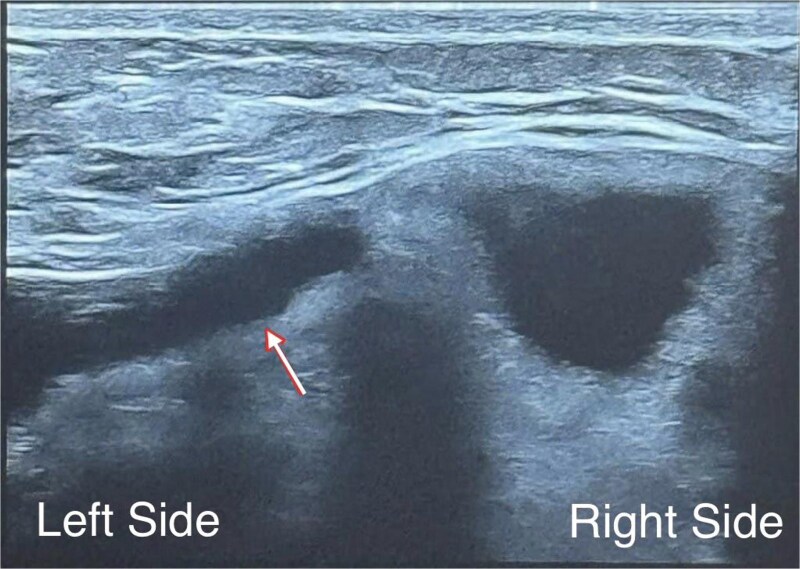
Diagnosis based on breast ultrasonography at 32 + 5 weeks’ gestation: We observed a multiloculated internal appearance (arrow), interpreted as intracapsular rupture, with no peri-implant seroma, extracapsular silicone, or regional lymphadenopathy; the right implant appeared intact.

Examination showed mild breast asymmetry without focal tenderness, skin changes, or clinically apparent peri-implant fluid or palpable axillary lymphadenopathy. Capsular contracture was assessed as Baker grade I, with a soft, pliable capsule. Ultrasound showed normal breast parenchyma bilaterally without suspicious masses and no abnormal axillary lymph nodes. The left implant demonstrated findings interpreted as intracapsular rupture, without evidence of extracapsular silicone or peri-implant seroma; the right implant appeared intact (Fig. 1).

After multidisciplinary discussion among obstetrician, radiologist, and plastic surgeon, a conservative antepartum management was chosen because the patient remained clinically stable and the imaging result was unlikely to change pregnancy management. Magnetic resonance imaging (MRI) was deferred. She continued routine obstetric follow-up and was given clear return precautions, including new unilateral swelling or rapid size change, increasing pain, erythema/warmth, fever, a new palpable mass, or axillary swelling.

**Figure 2:**
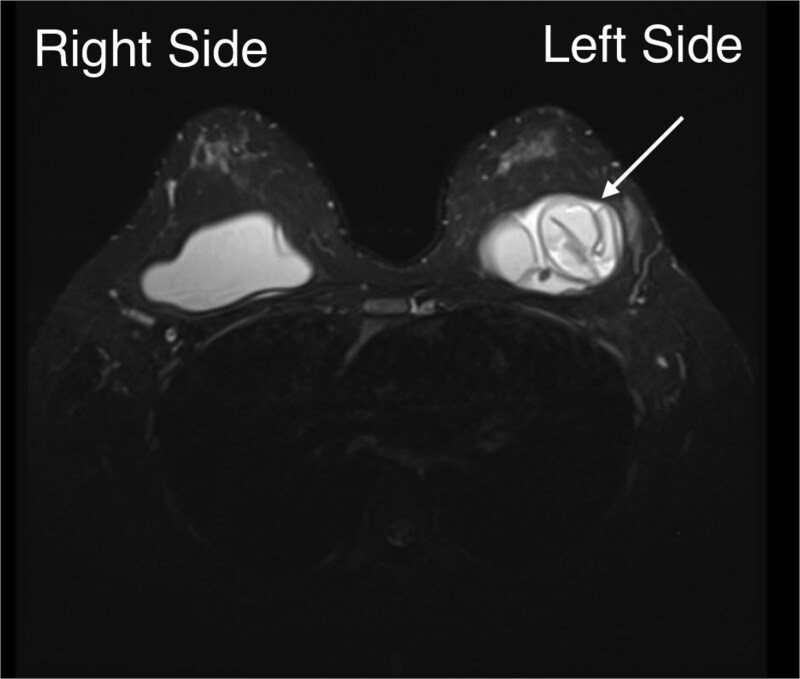
Postpartum breast MRI: The left implant shows the linguine sign represents intracapsular rupture and focal shell discontinuity in the upper half of the implant, with an associated subcapsular/peri-implant fluid collection measuring up to 24 mm. The right breast one shows an intact implant without shell discontinuity or peri-implant fluid collection.

She delivered by cesarean section at 38 weeks’ gestation after shared decision-making for obstetric/patient-preference reasons. She elected not to breastfeed and received lactation-suppression therapy. Postoperative MRI confirmed left implant rupture with intracapsular fluid, while the right implant and breast parenchyma were otherwise unremarkable, with only small benign-appearing axillary lymph nodes bilaterally (Fig. 2).

**Figure 3:**
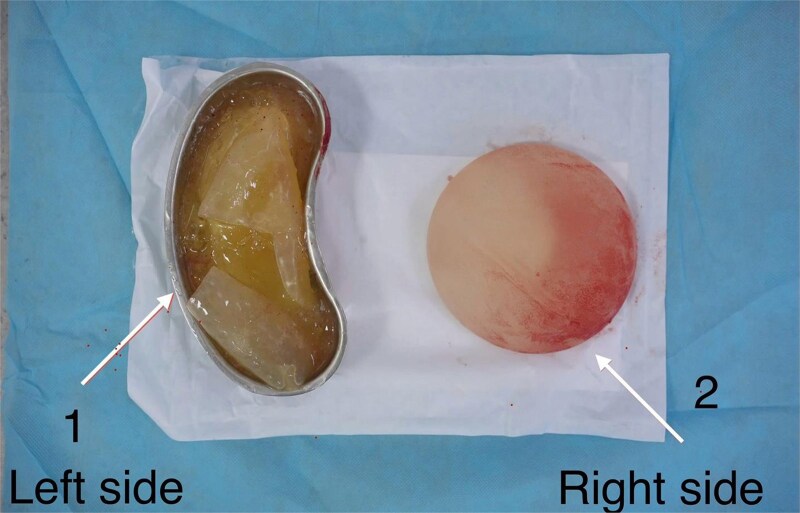
Intraoperative photograph of explanted breast implants: The left implant demonstrated intracapsular rupture, with turbid silicone–fluid admixture collected in a kidney dish (left side). The right implant appeared intact (right side).

At 7 months postpartum, elective surgery was performed using the prior inframammary incision. Bilateral pocket exploration confirmed left intracapsular rupture, with turbid silicone–fluid admixture encountered on explantation; the right one was intact (Fig. 3), (Supplementary Video S1). The capsule was clinically soft without marked thickening. The microbiology result then was unremarkable. Bilateral implant replacement with a 375-cc Textured Motiva Ergonomix implant was selected through shared decision-making, primarily to match the pre-existing volume and to address the patient’s preference to exchange an aged-ruptured textured implant. The recovery was uneventful.

## Discussion

In pregnancy, a suspected “implant rupture” may appear urgent despite a low objective clinical risk. In our case, the patient was asymptomatic, had only mild asymmetry with Baker I contracture, and ultrasound showed normal breast parenchyma without seroma, suspicious nodes, or extracapsular silicone—features supporting expectant management in gestation.

The cause of rupture is often hard to determine. Mechanisms described include iatrogenic damage at implantation, shell swelling, fold failure, and external trauma [5]. With an implant age of seven years and no history of trauma or manipulation, time-related shell fatigue represents a plausible explanation, whereas evidence that pregnancy directly causes rupture remains limited. Pregnancy is clinically important because physiologic breast enlargement and glandular proliferation can make clinical examination less specific [4, 5] and amplify concern about fetal safety and breastfeeding, increasing the likelihood of low-value escalation if a clear plan is not provided.

Observation is consistent with the natural history of silicone implant rupture. Silent rupture is common [2, 3], and MRI-based cohort data suggest most ruptures are intracapsular with relatively infrequent short-term progression to extracapsular rupture [6]. Ultrasound is appropriate as the standard first-line test in pregnancy and can suggest extracapsular (“snowstorm”) and intracapsular (“stepladder”) patterns. MRI is highly accurate for implant integrity [7]; in our case, antepartum MRI was deferred because it would not alter pregnancy care, while postpartum MRI confirmed intracapsular rupture and supported elective planning. Ultrasound is appropriate as the standard first-line test; MRI and other advanced imaging are best used selectively when findings would alter management [8].

Counseling should address common patient concerns regarding fetal safety and breastfeeding. Studies using silicon as a surrogate marker are generally reassuring, demonstrating no significant elevation in blood or breast-milk levels compared with controls [9–11]. Although silicone “bleed” may occur even with intact implants [12], biochemical assays have limited clinical utility for monitoring rupture or leakage [13, 14].

Based on this case, we propose a practical pregnancy-specific pathway: screen for red flags signs; use ultrasound for risk stratification; reassurance with symptom-triggered reassessment in low-risk cases; MRI can be postponed till postpartum unless in which scenarios where result would change management; and plan definitive treatment postpartum in an elective setting. Finally, women with implants should follow recommended routine surveillance of FDA, and those planning pregnancy may benefit from preconception assessment to reduce uncertainty during gestation.

## Supplementary Material

Video_1_rjag247

## References

[1] Spear SL, Murphy DK, Allergan Silicone Breast Implant U.S. Core Clinical Study Group. Natrelle round silicone breast implants: core study results at 10 years. Plast Reconstr Surg 2014;133:1354–61. 24867717 10.1097/PRS.0000000000000021 PMC4819531

[2] Dowden RV . Detection of gel implant rupture: a clinical test. Plast Reconstr Surg 1993;91:548–50. 8438029 10.1097/00006534-199303000-00025

[3] Hölmich LR, Fryzek JP, Kjøller K et al. The diagnosis of silicone breast-implant rupture: clinical findings compared with findings at magnetic resonance imaging. Ann Plast Surg 2005;54:583–9. 15900139 10.1097/01.sap.0000164470.76432.4f

[4] Katulski K, Katulski A, Nykowska A et al. Physiological changes in the mammary glands during a female’s life. Pol J Radiol 2024;89:e386–90. 39257924 10.5114/pjr/189566 PMC11384216

[5] Hillard C, Fowler JD, Barta R et al. Silicone breast implant rupture: a review. Gland Surg 2017;6:163–8. 28497020 10.21037/gs.2016.09.12 PMC5409893

[6] Hölmich LR, Friis S, Fryzek JP et al. Incidence of silicone breast implant rupture. Arch Surg 2003;138:801–6. 12860765 10.1001/archsurg.138.7.801

[7] Everson LI, Parantainen H, Detlie T et al. Diagnosis of breast implant rupture: imaging findings and relative efficacies of imaging techniques. AJR Am J Roentgenol 1994;163:57–60. 8010248 10.2214/ajr.163.1.8010248

[8] Chan Z, Laurence G, Mangubat E et al. Case 165: ruptured implant in pregnancy. In: Higgs MJ, Shiffman MA, (eds.), Cosmetic Breast Cases. Cham: Springer, 2016, 513–4.

[9] Semple JL, Lugowski SJ, Baines CJ et al. Breast milk contamination and silicone implants: preliminary results using silicon as a proxy measurement for silicone. Plast Reconstr Surg 1998;102:528–33. 9703094 10.1097/00006534-199808000-00038

[10] Peters W, Smith D, Lugowski S et al. Do patients with silicone-gel breast implants have elevated levels of blood silicon compared with control patients? Ann Plast Surg 1995;34:343–7. 7793777 10.1097/00000637-199504000-00001

[11] Peters W, Smith D, Lugowski S. Silicon assays in women with and without silicone gel breast implants--a review. Ann Plast Surg 1999;43:324–30. 10490190 10.1097/00000637-199909000-00020

[12] Winding O, Christensen L, Thomsen JL et al. Silicon in human breast tissue surrounding silicone gel prostheses. A scanning electron microscopy and energy dispersive X-ray investigation of normal, fibrocystic and peri-prosthetic breast tissue. Scand J Plast Reconstr Surg Hand Surg 1988;22:127–30. 3187446 10.3109/02844318809072383

[13] Bondurant S, Ernster V, Herdman R (eds). Safety of Silicone Breast Implants. Washington (DC): National Academies Press, 1999, Chapter 5. 20669503

[14] Bondurant S, Ernster V, Herdman R (eds). Safety of Silicone Breast Implants. Washington (DC): National Academies Press, 1999, Chapter 11. 20669503

